# The life‐course changes in muscle mass using dual‐energy X‐ray absorptiometry: The China BCL study and the US NHANES study

**DOI:** 10.1002/jcsm.13522

**Published:** 2024-07-01

**Authors:** Xi Wang, Liwang Gao, Jingfan Xiong, Hong Cheng, Li Liu, Hongbo Dong, Yiwen Huang, Hongmin Fan, Xia Wang, Xinying Shan, Pei Xiao, Junting Liu, Yinkun Yan, Jie Mi

**Affiliations:** ^1^ Center for Noncommunicable Disease Management Beijing Children's Hospital, Capital Medical University, National Center for Children's Health Beijing China; ^2^ School of Public Health Capital Medical University Beijing China; ^3^ Child and Adolescent Chronic Diseases Prevention and Control Department Shenzhen Center for Chronic Disease Control Shenzhen China; ^4^ Department of Epidemiology Capital Institute of Pediatrics Beijing China; ^5^ School of Public Health Guangdong Pharmaceutical University Guangzhou China; ^6^ North China University of Science and Technology Tangshan China; ^7^ Child Health Big Data Research Center Capital Institute of Pediatrics Beijing China

**Keywords:** China, dual‐energy X‐ray absorptiometry, muscle mass, NHANES, percentile curves

## Abstract

**Background:**

Sarcopenia is an important indicator of ill health and is linked to increased mortality and a reduced quality of life. Age‐associated muscle mass indices provide a critical tool to help understand the development of sarcopenia. This study aimed to develop sex‐ and age‐specific percentiles for muscle mass indices in a Chinese population and to compare those indices with those from other ethnicities using the National Health and Nutrition Examination Survey (NHANES) data.

**Methods:**

Whole‐body and regional muscle mass was measured by dual‐energy X‐ray absorptiometry (DXA) in participants of the China Body Composition Life‐course (BCL) study (17 203 healthy Chinese aged 3–60 years, male 48.9%) and NHANES (12 663 healthy Americans aged 8–59 years, male 50.4%). Age‐ and sex‐specific percentile curves were generated for whole‐body muscle mass and appendicular skeletal muscle mass using the Generalized Additive Model for Location Scale and Shape statistical method.

**Results:**

Values of upper and lower muscle mass across ages had three periods: an increase from age 3 to a peak at age 25 in males (with the 5th and 95th values of 41.5 and 66.4 kg, respectively) and age 23 in females (with the 5th and 95th values of 28.4 and 45.1 kg, respectively), a plateau through midlife (30s–50s) and then a decline after their early 50s. The age at which muscle mass began to decline was 52 years in men with the 5th and 95th percentile values of 43.5 and 64.6 kg, and 51 years in women with the 5th and 95th percentile values of 31.6 and 46.9 kg. Appendicular skeletal muscle mass decreased earlier than whole body muscle mass, especially leg skeletal muscle mass, which decreased slightly after age 49 years in both sexes. In comparison with their US counterparts in the NHANES, the Chinese participants had lower muscle mass indices (all *P* < 0.001) and reached a muscle mass peak earlier with a lower muscle mass, with the exception of similar values compared with adult Mexican and White participants. The muscle mass growth rate of Chinese children decreased faster than that of other races after the age of 13.

**Conclusions:**

We present the sex‐ and age‐specific percentiles for muscle mass and appendicular skeletal muscle mass by DXA in participants aged 3–60 from China and compare them with those of different ethnic groups in NHANES. The rich data characterize the trajectories of key muscle mass indices that may facilitate the clinical appraisal of muscle mass and improve the early diagnosis of sarcopenia in the Chinese population.

## Introduction

An aging population is a significant challenge for public health, with the elderly population increasing at an alarming rate in many countries around the world. According to census estimates for 2020, China and the United States had 13.5% and 16.7% of adults aged 65 and above, respectively, ranking in the top quarter of all countries and regions worldwide.^1^, ^2^ Age‐related loss of muscle mass and function, known as sarcopenia, is common in the elderly population, and the prevalence of low skeletal muscle mass in people aged 50–60 exceeds 10% in China and the United States (males <7.0 kg/m^2^ and females <5.4 kg/m^2^).^3^, ^4^, ^5^ In addition, sarcopenia combined with fat accumulation, known as sarcopenic obesity, is common in the aging population.^6^, ^7^ Low muscle mass and strength in childhood and adolescence are associated with increased risks for impairments in muscle parameters and cardiometabolic diseases later in life.^8^, ^9^ Understanding muscle development across the lifespan will be crucial for assessing the normal development and aging of muscle mass.

There remains a dearth of research on the developmental patterns of muscles throughout the entire lifespan in the Chinese population using dual‐energy X‐ray absorptiometry (DXA), and data are particularly lacking comparing muscle development disparities between the Chinese population and other ethnic groups across their life courses. Previous studies conducted in China investigating age‐related changes in muscle mass were limited in scale and focused solely on children or adults.^10^, ^11^, ^12^, ^13^ A study aimed at establishing grip strength standards for Chinese people aged 3–80 explored the trend of muscle index changes with age.^13^ However, because the research objective was not muscle development patterns and the use of bioelectrical impedance analysis technology to measure body composition has its shortcomings, characterizing the trajectories of muscle indices across the lifespan using DXA is desperately needed.

Several studies have reported the differences in free fat mass between races/ethnicities and sexes,^14^, ^15^, ^16^ but whether these sex and race/ethnicity differences in muscle mass vary at different age periods remains unknown. It is of great significance to understand racial differences in cardiovascular metabolic abnormalities, as muscle has a significant impact on cardiovascular metabolism.^5^ Prior studies using data from the National Health and Nutrition Examination Survey (NHANES) 1999–2004 described the age‐related changes in body composition, including lean mass, by sex and race/ethnicity from childhood to adulthood^17^, ^18^, ^19^; however, these studies did not include Asian individuals. In addition, the prior NHANES studies did not exclude participants with cardiovascular and metabolic diseases, which may lead to an inaccurate estimation of age‐specific muscle mass.

Body composition can be measured through a variety of techniques, such as computed tomography (CT), magnetic resonance imaging (MRI), air displacement plethysmography (ADP) and DXA. While CT, MRI and ADP offer the highest accuracy and precision, they are costly and inconvenient for clinical purposes.^20^ DXA is a practical option that accurately quantifies muscle mass and fat mass (as well as bone mineral content) and provides total and regional values for these parameters.^21^, ^22^ Therefore, the primary aim of this study was to construct sex‐ and age‐specific reference values based on healthy children and adults in China, measured by DXA. We also compared the sex and age curves of muscle mass between different ethnic groups in China and the United States, which may help better understand the racial differences in body components.

## Material and methods

### Study sample

The participants were recruited from the China Body Composition Life‐course (BCL) study from 2013 to 2023. BCL is a nationwide, ongoing population‐based cross‐sectional study involving nine urban areas (i.e., Beijing, Tianjin, Tangshan, Changchun, Jinan, Yinchuan, Shanghai, Chongqing and Guangzhou) in China, which was designed to understand patterns of body composition change over the life course of the well‐nourished Chinese population. The first round of the BCL study was conducted from 2013 to 2019, recruiting a sample of 13 399 children and adolescents aged 3–17 years from selected schools in each city. In April 2021, the study began to enrol healthy young and middle‐aged adults from the staff of universities, research institutions and hospitals in the selected cities. Up to August 2023, 5443 adults aged 18–60 years participated and underwent DXA scans. In September 2023, the BCL study entered a new phase: the enrolment of healthy elderly subjects aged over 60 years old (ongoing). For Americans, a total of 59 804 participants were examined from the NHANES between 2011 and 2018, and the details of the study methods are available elsewhere.^23^

**Figure 1:**
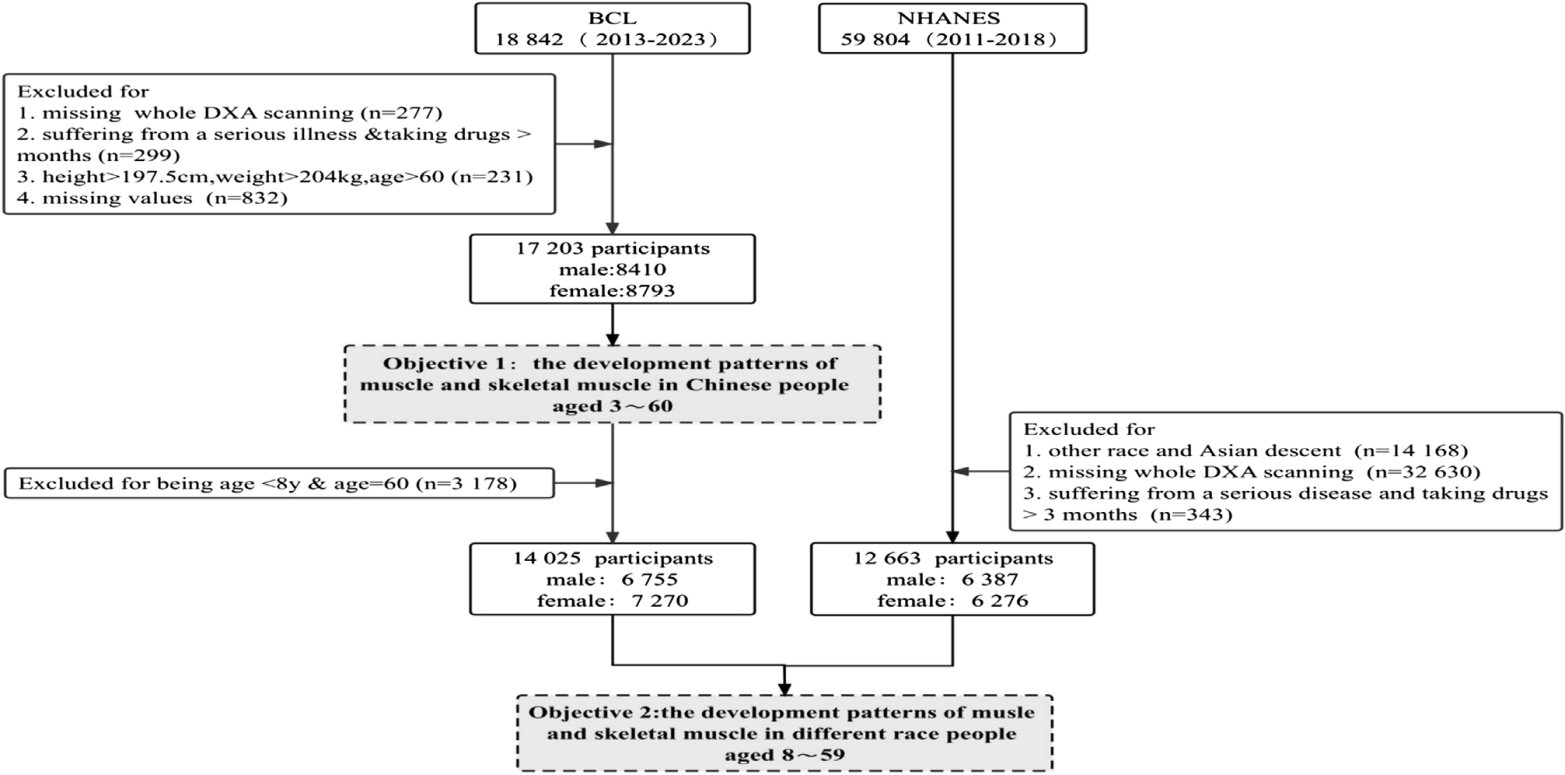
Flow chart detailing the selection process for participants. BCL, China Body Composition Life‐course study; DXA, dual‐energy X‐ray absorptiometry; NHANES, National Health and Nutrition Examination Survey.

To ensure that the body muscle mass patterns reflect the biological variation in a disease‐free population, we further excluded individuals who met the following seven exclusion criteria: (1) incomplete information on body composition; (2) any condition or use of any drug known to affect body composition indices; (3) nonremovable objects (e.g., prostheses, implants or casts); (4) body weight > 204 kg, height > 197.5 cm or weight or height beyond the measurement range of machines; (5) participation in research involving ionizing radiation in the past year; (6) some chronic diseases; and (7) pregnancy. Ultimately, 17 203 Chinese participants aged 3–60 years (8410 males and 8793 females) were used to develop the patterns of muscle mass parameters. In addition, 12 663 (6276 males and 6387 females) from NHANES aged 8–59 years were used to compare the muscle mass indices in those different ethnicities (*Figure* *1*).

### Anthropometry and body composition measurement

In BCL, wall‐mounted stadiometers and beam scales were used to measure standing height and weight, respectively. Body composition was measured using whole‐body DXA (Hologic, Bedford, MA, USA) by a trained technician according to a standardized protocol. Data from DXA included values for the fat mass (grams), bone mineral content (grams) and lean mass (grams) of the whole body. Muscle mass was calculated as the whole lean mass minus the whole bone mineral content. Appendicular skeletal muscle mass (ASMM) was calculated as the total limb lean mass minus the total limb bone mineral content, which was a good proxy for whole‐body skeletal muscle mass,^24^ and arm skeletal muscle mass (arm‐SMM) and leg skeletal muscle mass (leg‐SMM) were also calculated by subtracting the bone mineral content from the limb lean mass of the corresponding parts. Muscle mass index (MMI; whole muscle mass/height^2^), appendicular skeletal muscle mass index (ASMMI; ASMM/height^2^), arm skeletal muscle mass index (arm‐SMMI; arm‐SMM/height^2^) and leg skeletal muscle mass index (leg‐SMMI; leg‐SMM/height^2^) were also calculated.

In the NHANES, height and weight were measured by trained health technicians using standardized protocols. Body composition indicators were measured using Hologic fan‐beam densitometers (Hologic) under a standardized protocol. Muscle mass indices were calculated using the same indicators and formulas as in the BCL.

### Statistical analysis

Continuous variables were expressed as the mean ± standard deviation (SD) according to their distribution type, and an analysis of variance (ANOVA) was conducted to compare the general characteristics among the ethnic groups in males and females.

Age‐ and sex‐specific percentile curves for muscle mass indices were generated using the Generalized Additive Model for Location Scale and Shape (GAMLSS) model for the Box–Cox power exponential (BCPE) or Box–Cox t (BCT) distribution with cubic spline smoothing. Both distributions have four parameters, including μ, σ, ν and τ, which represent location (median), scale (approximate coefficient of variation), skewness (power transformation to symmetry) and kurtosis (degrees of freedom or power exponential parameter), respectively. The goodness of fit of the models was assessed by the Bayesian information criterion and by Q–Q plots. The final best models were based on the BCT distribution for muscle mass in males and females and on the BCPE distribution for ASMM. The reference values of muscle mass parameter percentiles (5th, 50th and 95th) were computed by age, and smoothed ethnic‐specific median muscle mass parameter curves were also modelled simultaneously to examine the average difference in muscle mass indices between four ethnicities (Chinese, non‐Hispanic White, non‐Hispanic Black and Mexican American) aged 8–59 years using data from BCL and NHANES. In addition, we estimated the age‐specific change rates of muscle mass parameters by using the first derivatives of the median trajectories.

Data analyses were performed using the GAMLSS 4.3‐1 library running under R 4.1.2 (R Foundation for Statistical Computing, Vienna, Austria).

## Results

### Characteristics of subjects

A maximum of 14 797 males (8410 Chinese from the BCL study, 2851 non‐Hispanic White, 2030 non‐Hispanic Black and 1506 Mexican American) and 15 069 females (8793 Chinese from the BCL study, 2777 non‐Hispanic White, 1928 non‐Hispanic Black and 1571 Mexican American) were included (*Table* *1*). Non‐Hispanic White males were tallest, and weight was lowest in Chinese individuals. MMI levels were highest in non‐Hispanic White males and non‐Hispanic Black females compared with other ethnicities (all *P* < 0.001). MMI indices were highest in non‐Hispanic Black males and lowest in Chinese, and the results were the same in females. The age composition is shown in *Table* *S1*.

**Table 1:** Characteristics of the participants in China from the China Body Composition Life‐course study and the United States from the National Health and Nutrition Examination Survey

	Men					Women				
	Chinese	Non‐Hispanic Whites	Non‐Hispanic Blacks	Mexican Americans	*P* ^a^	Chinese	Non‐Hispanic Whites	Non‐Hispanic Blacks	Mexican Americans	*P* ^a^
Age (years)	15.0 ± 9.5	30.1 ± 15.8	27.4 ± 15.9	26.3 ± 15.1	<0.001	16.9 ± 11.3	31.1 ± 15.7	29.1 ± 16.4	26.8 ± 15.6	<0.001
Height (m)	153.8 ± 22.4	170.2 ± 14.6	168.5 ± 14.9	164.1 ± 14.0	<0.001	149.0 ± 17.9	160.5 ± 10.3	159.4 ± 10.4	154.0 ± 10.0	<0.001
Weight (kg)	51.1 ± 22.0	77.7 ± 26.4	75.6 ± 28.0	74.1 ± 25.0	<0.001	45.4 ± 16.0	70.0 ± 23.2	73.6 ± 26.1	66.0 ± 22.4	<0.001
BMI (kg/m^2^)	20.4 ± 4.7	26.1 ± 6.8	25.9 ± 7.4	26.8 ± 6.8	<0.001	19.7 ± 4.0	26.8 ± 7.7	28.5 ± 8.7	27.3 ± 7.7	<0.001
Muscle parameter										
Muscle mass (kg)	36.2 ± 15.3	51.4 ± 15.5	51.4 ± 16.7	48.2 ± 15.0	<0.001	29.3 ± 9.6	39.1 ± 10.1	41.1 ± 11.4	36.3 ± 10.1	<0.001
Muscle mass index (kg/m^2^)	14.4 ± 3.0	17.3 ± 3.6	17.6 ± 3.9	17.4 ± 3.8	<0.001	12.7 ± 2.1	15.0 ± 3.1	15.9 ± 3.5	15.1 ± 3.2	<0.001
Appendicular muscle mass (kg)	16.2 ± 7.4	23.5 ± 7.3	25.1 ± 8.3	22.1 ± 7.0	<0.001	12.4 ± 4.4	17.0 ± 4.5	19.1 ± 5.5	15.5 ± 4.4	<0.001
Appendicular muscle mass index (kg/m^2^)	6.3 ± 1.7	7.9 ± 1.7	8.6 ± 2.0	8.0 ± 1.8	<0.001	5.3 ± 1.1	6.5 ± 1.4	7.4 ± 1.7	6.5 ± 1.4	<0.001
Arm muscle mass (kg)	3.8 ± 1.8	6.4 ± 2.2	6.7 ± 2.5	6.0 ± 2.2	<0.001	2.7 ± 0.9	3.9 ± 1.0	4.4 ± 1.3	3.7 ± 1.1	<0.001
Arm muscle mass index (kg/m^2^)	1.5 ± 0.4	2.1 ± 0.6	2.3 ± 0.6	2.16 ± 0.6	<0.001	1.2 ± 0.3	1.5 ± 0.3	1.7 ± 0.4	1.6 ± 0.4	<0.001
Leg muscle mass (kg)	12.4 ± 5.7	17.1 ± 5.2	18.4 ± 5.9	16.0 ± 5.0	<0.001	9.7 ± 3.5	13.0 ± 3.6	14.7 ± 4.3	11.8 ± 3.4	<0.001
Leg muscle mass index (kg/m^2^)	4.9 ± 1.3	5.8 ± 1.2	6.3 ± 1.4	5.8 ± 1.2	<0.001	4.2 ± 0.9	5.0 ± 1.1	5.7 ± 1.3	4.9 ± 1.1	<0.001

Abbreviation: BMI, body mass index.

^a^ *P* value: difference between ethnic groups and Chinese participants.

### Age‐related changes in muscle mass parameters in Chinese individuals by sex

**Figure 2:**
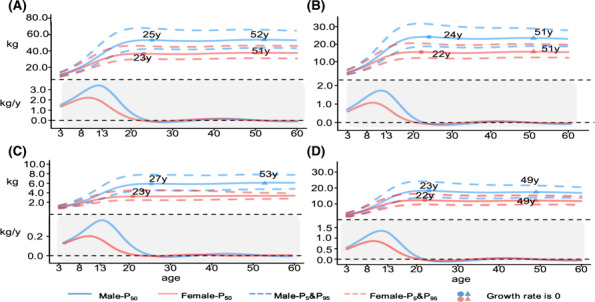
Reference percentiles and age‐specific change rates in Chinese children and adults by sex for muscle mass (A), appendicular skeletal muscle mass (B), arm skeletal muscle mass (C) and leg skeletal muscle mass (D). Age‐specific change rates of muscle mass parameters were calculated by using the first derivatives of the median trajectories.^25^

The overall trajectories in total muscle mass were similar between sexes. In general, muscle mass increased from childhood, peaked during the mid‐20s, plateaued in middle adulthood (30–50 years) and then decreased steadily throughout late adulthood. From the age of 3 to 10, boys and girls had a similar median level (5th, 95th) of muscle mass, which increased from 11.2 (9.1, 14.1) and 10.7 (8.4, 14.0) to 26.5 (20.1, 36.4) and 23.4 (18.3, 31.4), respectively, with males maintaining a comparatively higher level throughout life. It peaked at 25 years of age for males (54.3 [41.5, 66.4]) and 23 years of age for females (36.7 [28.4, 45.1]). The growth rate remained positive until reaching its peak and then continued to fluctuate until it began to decline at the age of 52 for males and 51 for females (*Figure* *2* *A*). *Figure* *2* *B–D* presents the ASMM, arm‐SMM and leg‐SMM percentile curves for males and females and shows similarities in the shape of muscle mass. However, the ASMM, especially leg‐SMM, peaked early and decreased earlier than total muscle mass in both males and females, while the arm‐SMM did not decrease after the age of 50. For males, the 50th percentile of leg‐SMM peaked at 18.3 (14.1, 24.0) at the age of 23 years and declined rapidly beginning at age 49. Leg‐SMM peaked at 12.3 (9.6, 16.2) for females at age 22 but declined rapidly beginning at age 49. Percentile curves for MMI, ASMMI, arm‐SMMI and leg‐SMMI are available in *Figure* *S1*.

The 5th, 50th and 95th percentiles of muscle mass parameters for males and females at specific ages are also presented in *Table* *2*. Compared with age 50, the median percentiles of muscle mass, ASMM and leg‐SMM declined by 0.6, 0.5 and 0.5 kg in males and by 0.3, 0.2 and 0.3 kg in females at age 60, respectively. However, there was no decrease in arm‐SMM between men and women aged 60 compared with those aged 50.

**Table 2:** Percentiles (5th, 50th and 95th) of muscle mass parameters by sex for Chinese children and adults at specific ages

Age	MM			ASMM			Arm‐SMM			Leg‐SMM		
	5th	50th	95th	5th	50th	95th	5th	50th	95th	5th	50th	95th
Males												
5	11.4	14.4	18.6	3.9	5.4	7.4	1.0	1.4	1.8	2.8	4.1	5.6
10	20.1	26.5	36.4	8.4	11.7	16.3	1.9	2.6	3.6	6.4	9.1	12.8
15	32.6	42.8	57.9	14.7	19.9	27.3	3.3	4.5	6.2	11.4	15.4	21.3
20	40.9	51.9	67.4	18.3	23.8	31.6	4.3	5.6	7.6	13.9	18.2	24.2
30	42.1	52.5	66.2	18.2	23.4	30.3	4.5	5.9	7.8	13.6	17.5	22.8
40	42.3	52.4	65.3	18.2	23.1	29.3	4.5	5.9	7.7	13.5	17.2	21.8
50	43.7	53.7	66.0	18.9	23.5	29.0	4.8	6.1	7.8	13.9	17.4	21.5
60	43.3	53.1	64.7	18.7	23.0	27.8	4.8	6.1	7.8	13.6	16.9	20.4
Females												
5	10.6	13.5	17.9	3.7	5.1	7.3	0.9	1.2	1.7	2.7	4.0	5.7
10	18.3	23.4	31.4	7.3	9.9	13.8	1.6	2.1	3.0	5.6	7.8	10.9
15	26.2	32.9	43.1	10.9	14.3	19.4	2.2	3.0	4.1	8.5	11.3	15.4
20	29.6	36.4	46.3	12.1	15.5	20.6	2.4	3.2	4.5	9.5	12.3	16.3
30	29.7	36.1	45.0	11.8	15.0	19.6	2.4	3.2	4.5	9.2	11.8	15.3
40	30.5	37.0	45.7	12.1	15.3	19.8	2.5	3.3	4.4	9.4	11.8	15.2
50	31.1	37.8	46.5	12.4	15.7	20.1	2.7	3.3	4.0	9.6	12.0	15.3
60	30.7	37.5	46.2	12.3	15.4	19.7	2.8	3.4	3.9	9.4	11.8	14.8

Abbreviations: ASMM, appendicular skeletal muscle mass; MM, muscle mass; SMM, skeletal muscle mass.

### Comparisons in muscle mass parameters in Chinese, non‐Hispanic White, non‐Hispanic Black and Mexican American individuals

**Figure 3:**
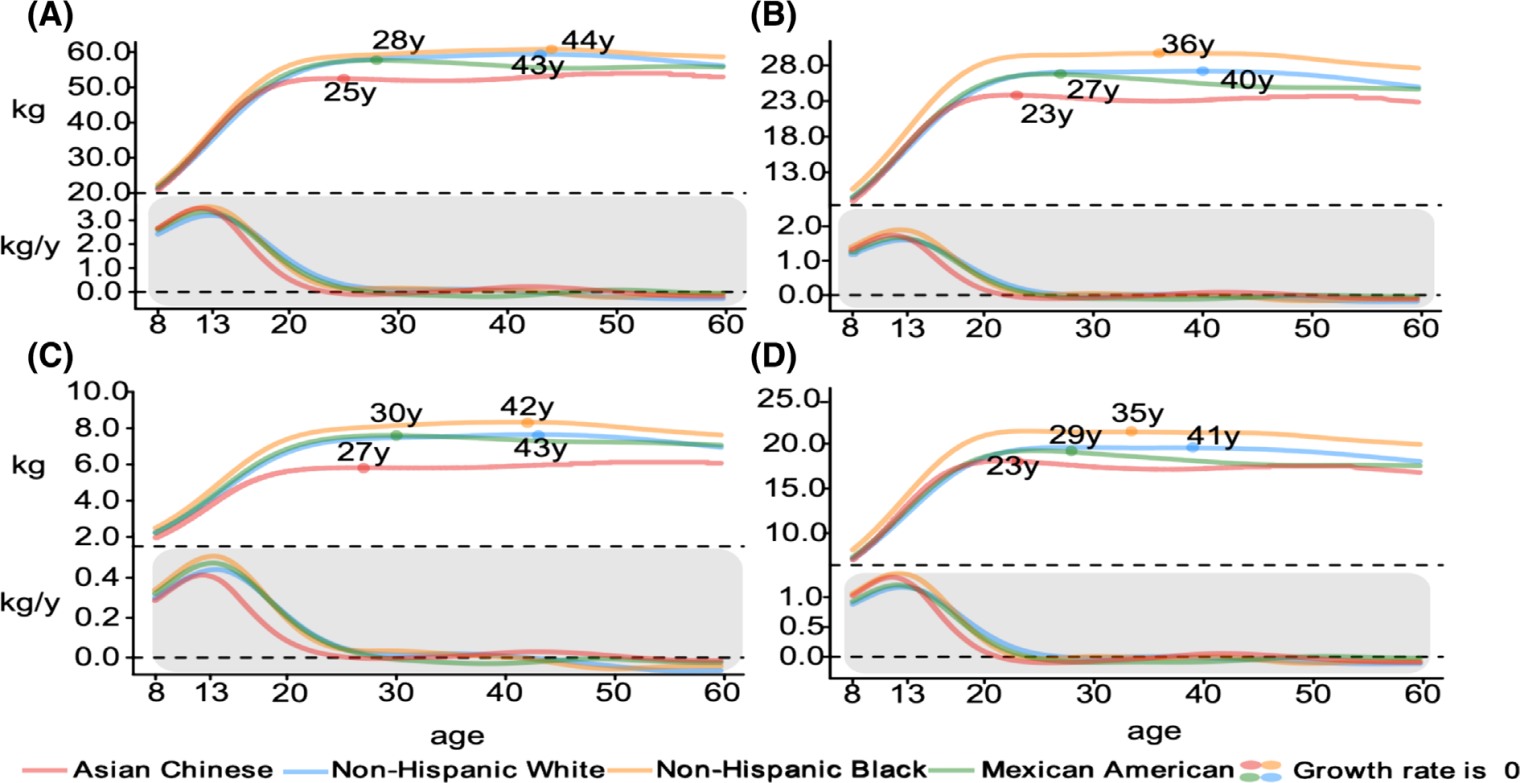
Comparisons of the 50th percentile curves and change rates at different ages in Chinese (from the China Body Composition Life‐course study) versus American (White, Black and Mexican from the NHANES) male participants for muscle mass (A), appendicular skeletal muscle mass (B), arm skeletal muscle mass (C) and leg skeletal muscle mass (D). The age‐specific change rates of muscle mass parameters were calculated by using the first derivatives of the median trajectories.^25^

**Figure 4:**
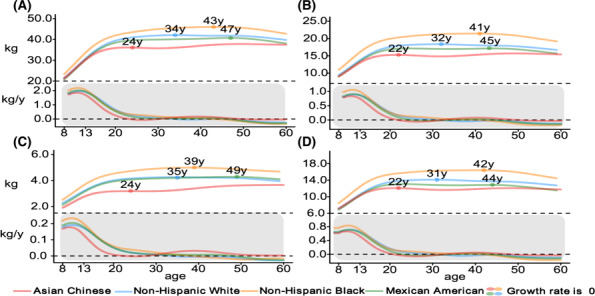
Comparisons of the 50th percentile curves and change rates at different ages in Chinese (from the China Body Composition Life‐course study) versus American (White, Black and Mexican from the NHANES) female participants for muscle mass (A), appendicular skeletal muscle mass (B), arm skeletal muscle mass (C) and leg skeletal muscle mass (D). The age‐specific change rates of muscle mass parameters were calculated by using the first derivatives of the median trajectories.^25^

Comparisons with the ethnic groups (including non‐Hispanic Whites, non‐Hispanic Blacks and Mexican Americans) of US counterparts from the NHANES reference data for the 50th percentile curves of muscle mass, ASMM, arm‐SMM and leg‐SMM are shown in *Figures* *3* and *4*. Compared with the White, Black and Mexican populations in the United States, the 50th percentiles of muscle mass indices were similar in children but consistently and distinctly lower in Chinese adults. The muscle mass growth rate of Chinese children decreased faster than that of other races after the age of 13, and the muscle peak age of Chinese children was younger than that of the other three races.

The differences in the percentage curves of MMI, ASMMI, arm‐SMMI and leg‐SMMI among races are similar to those for muscle mass, as shown in *Figures* *S2* and *S3*.

## Discussion

The study used DXA data to estimate the normative trajectories of muscle mass indices by sex from the BCL from China and compared these trajectories in other race groups in the United States from the NHANES. First, the results characterized revealed critical time points when muscle mass indices changed trajectories between ages 3 and 60. Total muscle mass increased in childhood, peaked during the mid‐20s and decreased steadily after age 50. More interestingly, the main characteristic of the decline stage was a decrease in leg muscle mass, which means that as age increases, leg muscles age first. Such information may help monitor muscle mass loss in both children and adults. Second, in comparison with White, Black and Mexican populations in the United States, the Chinese population reached muscle mass peak earlier and had lower muscle mass than their US counterparts, despite similar muscle mass before the age of 10 years. In particular, Chinese children had a greater growth rate in the age range of 8–13 years in leg‐SMM, but the growth rate decreased rapidly in the age range of 13–25 years.

Age‐related changes in lean mass (including muscle mass and bone mass) or muscle mass have been developed in various regions worldwide, including India,^26^ Austria,^27^ Mexico,^28^ Australia,^29^ China^11^ and America.^30^ However, most of these studies had small sample sizes, involved either children or adults but not both or used less accurate methods to measure muscle mass.^26^, ^27^, ^28^, ^29^ For example, in studies of Indian, Austrian and Mexican children and adolescents,^26^, ^27^, ^28^ DXA‐derived total muscle mass and appendicular skeletal muscle mass indices showed an increasing trend with age in early life for both males and females. A study on adults in western China showed that the skeletal muscle mass index (SMMI) measured by bioelectrical impedance analysis decreased with age from 45 to 55 years.^11^ Our data provide important extensions to these findings by directly constructing age‐specific muscle mass trajectories from childhood to adulthood. Consistent with previous studies,^31^, ^32^ our data demonstrated a similar age‐related change trend in which muscle mass increased rapidly from childhood and progressively decreased in late adulthood for both sexes. In addition, in line with previous findings,^33^ the current study demonstrated sex differences in muscle mass throughout the lifespan, with males having greater muscle mass than females. These differences also underscore the need to establish separate reference values for muscle mass for males and females.

Specifically, we estimated the change rate of muscle mass using GAMLSS and further identified its peak age and the age at which it began to decrease. We found that muscle mass reached its peak at approximately 25 years for males and 23 years for females, and males versus females showed greater increase rates from age 8 years to peak age. The sex disparity in muscle mass may be partly explained by different levels of hormones,^34^, ^35^, ^36^ such as testosterone, which have been shown to be associated with muscle mass gains. In addition, our study showed that muscle mass remained stable until the age of 50, after which it began to decrease in both sexes, and ASMM, especially leg‐SMM, decreased earlier in both sexes than total muscle mass. This is consistent with previous studies in Chinese adults,^13^ which have shown that SMMI begins to decline between the ages of 45 and 55.

Previous studies have shown that Asians have a greater risk for cardiovascular risk factors than Whites at similar body weight status.^37^, ^38^ Muscle mass differences may partially underlie the differences in cardiovascular risk between Asians and American Whites. In the current study, we examined ethnic differences in muscle mass and found that, compared with American counterparts in the NHANES data, the values of muscle mass and ASMM were distinctly lower in Chinese males and females. An interesting finding was that in both sexes, Chinese children had a consistent or even higher change rate compared with other races but peaked at an earlier age in youth, indicating that the growth potential of muscle mass in Chinese people is lower than that of other races. The race/ethnicity differences in lean mass may be largely driven by genetic differences.^39^, ^40^ No prior studies were conducted in this regard, and further research is needed to confirm our findings and explore the underlying mechanisms.

### Strengths and limitations

The strengths of the present study include the large sample size, inclusion of multiple ethnic groups, broad age range and use of well‐validated and accurate techniques to quantify muscle mass. Our study has several limitations. First, our study was limited by its cross‐sectional design and cannot accurately assess age‐related changes in muscle mass at the individual level. Second, the number of Chinese participants older than 50 years was low (*n* = 296, 1.72%), which can affect the validity of the reference range in this age group. Third, we did not develop age‐related changes for those older than 60 years. We are expanding our study to those older participants. Finally, our adulthood sex‐ and age‐specific percentiles were developed from professional men and women with a high health literacy. This introduces the possibility of selection bias in adult individuals seeking body composition assessments.

## Conclusions

To conclude, we have developed normative trajectories for total and regional muscle mass parameters for Chinese population aged 3–60 years in a large nationwide cross‐sectional study, which will improve the understanding of growth and aging processes of muscle mass. Additionally, this survey also provides insights into the differences in muscle development patterns among different ethnic groups. Further research will be needed to examine the important determinants of muscle mass at different life stages and the factors that contribute to racial differences in muscle mass.

## Conflict of interest statement

The authors declare that they have no competing interests.

## Supporting information


**Table S1.** Age composition of the participants
**Figure S1.** Muscle mass index (MMI) (A), appendicular skeletal muscle mass index (ASMMI) (B), arm skeletal muscle mass index (arm‐SMMI) (C) and leg skeletal muscle mass index (leg‐SMMI)(D) reference percentiles for males and females between ages 3 to 65 years in Chinese
**Figure S2.** Comparisons of the 50^th^ percentile curves for muscle mass index (MMI) (A), appendicular skeletal muscle mass index (ASMMI) (B), arm skeletal muscle mass index (arm‐SMMI) (C) and leg skeletal muscle mass index (leg‐SMMI)(D) in males according to age for Chinese versus American (including white, black, and Mexican adults) from NHANES data
**Figure S3.** Comparisons of the 50^th^ percentile curves for muscle mass index (MMI) (A), appendicular skeletal muscle mass index (ASMMI) (B), arm skeletal muscle mass index (arm‐SMMI) (C) and leg skeletal muscle mass index (leg‐SMMI)(D) in females according to age for Chinese versus American (including white, black, and Mexican adults) from NHANES data

## References

[1] National Bureau of Statistics . Communiqu é of the Seventh National Population Census. 2020.

[2] United States Census Bureau . 2020 census results. Available from: https://www.census.gov/library/stories/2023/05/2020‐census‐united‐states‐older‐population‐grew.html. Accessed 02 Aug 2023.

[3] Cruz‐Jentoft AJ , Sayer AA . Sarcopenia. Lancet 2019;393:2636–2646. 31171417 10.1016/S0140-6736(19)31138-9

[4] Gandham A , Mesinovic J , Jansons P , Zengin A , Bonham MP , Ebeling PR , et al. Falls, fractures, and areal bone mineral density in older adults with sarcopenic obesity: a systematic review and meta‐analysis. Obes Rev 2021;22:e13187. 33491333 10.1111/obr.13187

[5] Izzo A , Massimino E , Riccardi G , Della PG . A narrative review on sarcopenia in type 2 diabetes mellitus: prevalence and associated factors. Nutrients 2021;13:183. 33435310 10.3390/nu13010183 PMC7826709

[6] Batsis JA , Villareal DT . Sarcopenic obesity in older adults: etiology, epidemiology and treatment strategies. Nat Rev Endocrinol 2018;14:513–537. 30065268 10.1038/s41574-018-0062-9 PMC6241236

[7] Stewart CE , Sharples AP . Aging, skeletal muscle, and epigenetics. Plast Reconstr Surg 2022;150:27–33. 10.1097/PRS.0000000000009670 36170433

[8] García‐Hermoso A , Ramírez‐Campillo R , Izquierdo M . Is muscular fitness associated with future health benefits in children and adolescents? A systematic review and meta‐analysis of longitudinal studies. Sports Med 2019;49:1079–1094. 30953308 10.1007/s40279-019-01098-6

[9] Orsso CE , Tibaes JRB , Oliveira CLP , Rubin DA , Field CJ , Heymsfield SB , et al. Low muscle mass and strength in pediatrics patients: why should we care? Clin Nutr 2019;38:2002–2015. 31031136 10.1016/j.clnu.2019.04.012

[10] Liu J , Yan Y , Xi B , Huang G , Mi J . Skeletal muscle reference for Chinese children and adolescents. J Cachexia Sarcopenia Muscle 2019;10:155–164. 30499245 10.1002/jcsm.12361 PMC6438334

[11] Zhang J , Li J , Chen C , Yin T , Wang Q , Li X , et al. Reference values of skeletal muscle mass, fat mass and fat‐to‐muscle ratio for rural middle age and older adults in western China. Arch Gerontol Geriatr 2021;95:104389. 33713879 10.1016/j.archger.2021.104389

[12] Jin M , Du H , Zhang Y , Zhu H , Xu K , Yuan X , et al. Characteristics and reference values of fat mass index and fat free mass index by bioelectrical impedance analysis in an adult population. Clin Nutr 2019;38:2325–2332. 30389251 10.1016/j.clnu.2018.10.010

[13] He H , Pan L , Wang D , Liu F , Du J , Pa L , et al. Normative values of hand grip strength in a large unselected Chinese population: evidence from the China National Health Survey. J Cachexia Sarcopenia Muscle 2023;14:1312–1321. 36999522 10.1002/jcsm.13223 PMC10235885

[14] Nickerson BS , Tinsley GM , Fedewa MV , Esco MR . Fat‐free mass characteristics of Hispanic adults: comparisons with non‐Hispanic Caucasians and cadaver reference values. Clin Nutr 2020;39:3080–3085. 32057536 10.1016/j.clnu.2020.01.013

[15] Blue MNM , Tinsley GM , Ryan ED , Smith‐Ryan AE . Validity of body‐composition methods across racial and ethnic populations. Adv Nutr 2021;12:1854–1862. 33684215 10.1093/advances/nmab016 PMC8528114

[16] Shypailo RJ , Wong WW . Fat and fat‐free mass index references in children and young adults: assessments along racial and ethnic lines. Am J Clin Nutr 2020;112:566–575. 32469402 10.1093/ajcn/nqaa128

[17] Borrud LG , Flegal KM , Looker AC , Everhart JE , Harris TB , Shepherd JA . Body composition data for individuals 8 years of age and older: U.S. population, 1999–2004. Vital Health Stat 2010;11:1–87. PMC5901781 20812448

[18] Hinton BJ , Fan B , Ng BK , Shepherd JA . Dual energy X‐ray absorptiometry body composition reference values of limbs and trunk from NHANES 1999–2004 with additional visualization methods. PLoS ONE 2017;12:e0174180. 28346492 10.1371/journal.pone.0174180 PMC5367711

[19] Kelly TL , Wilson KE , Heymsfield SB . Dual energy X‐ray absorptiometry body composition reference values from NHANES. PLoS ONE 2009;4:e7038. 19753111 10.1371/journal.pone.0007038 PMC2737140

[20] Buckinx F , Landi F , Cesari M , Fielding RA , Visser M , Engelke K , et al. Pitfalls in the measurement of muscle mass: a need for a reference standard. J Cachexia Sarcopenia Muscle 2018;9:269–278. 29349935 10.1002/jcsm.12268 PMC5879987

[21] Huang Y , Dong H , Cheng H , Shan X , Yu X , Xie X , et al. Differences in body composition measurements assessed by air displacement plethysmography and dual‐energy X‐ray absorptiometry in young and middle‐aged adults. Clin Nutr ESPEN. 2022;50:111–117. 35871911 10.1016/j.clnesp.2022.06.014

[22] Huang Y , Wang X , Cheng H , Dong H , Shan X , Zhao X , et al. Differences in air displacement plethysmography, bioelectrical impedance analysis and dual‐energy X‐ray absorptiometry for estimating body composition in Chinese children and adolescents. J Paediatr Child Health 2023;59:470–479. 36661380 10.1111/jpc.16327

[23] Centers for Disease Control and Prevention . National Health and Nutrition Examination Survey. Available from: https://www.cdc.gov/nchs/nhanes/index.htm. Accessed 20 Jun 2023.

[24] Kim J , Wang Z , Heymsfield S , Baumgartner R , Gallagher D , et al. Total‐body skeletal muscle mass: estimation by a new dual‐energy X‐ray absorptiometry method. Am J Clin Nutr 2020;76:378–383. 10.1093/ajcn/76.2.378 12145010

[25] Stasinopoulos D , Rigby A . Generalized additive models for location scale and shape (GAMLSS) in R. J Stat Softw 2007;23:1–46.

[26] Van Beijsterveldt I , van der Steen M , de Fluiter KS , Spaans S , Hokken‐Koelega ACS , et al. Body composition and bone mineral density by dual energy x‐ray absorptiometry: reference values for young children. Clin Nutr 2022;41:71–79. 34864456 10.1016/j.clnu.2021.11.010

[27] Ofenheimer A , Breyer‐Kohansal R , Hartl S , Burghuber OC , Krach F , Schrott A , et al. Reference charts for body composition parameters by dual‐energy X‐ray absorptiometry in European children and adolescents aged 6 to 18 years—results from the Austrian LEAD (Lung, hEart, sociAl, boDy) cohort. Pediatr Obes 2021;16:e12695. 32618143 10.1111/ijpo.12695 PMC7757249

[28] Clark P , Denova‐Gutiérrez E , Ambrosi R , Szulc P , Rivas‐Ruiz R , Salmerón J . Reference values of total lean mass, appendicular lean mass, and fat mass measured with dual‐energy X‐ray absorptiometry in a healthy Mexican population. Calcif Tissue Int 2016;99:462–471. 27484026 10.1007/s00223-016-0181-z

[29] Kirk B , Bani Hassan E , Brennan‐Olsen S , Vogrin S , Bird S , Zanker J . Body composition reference ranges in community‐dwelling adults using dual‐energy X‐ray absorptiometry: the Australian Body Composition (ABC) Study. J Cachexia Sarcopenia Muscle 2021;12:880–890. 33991068 10.1002/jcsm.12712 PMC8350202

[30] Kindler JM , Kalkwarf HJ , Lappe JM , Gilsanz V , Oberfield S , Shepherd JA , et al. Pediatric reference ranges for ultradistal radius bone density: results from the Bone Mineral Density in Childhood Study. J Clin Endocrinol Metab 2020;105:e3529–e3539. 32561914 10.1210/clinem/dgaa380 PMC7465545

[31] McCarthy HD , Samani‐Radia D , Jebb SA , Prentice AM . Skeletal muscle mass reference curves for children and adolescents. Pediatr Obes 2014;9:249–259. 23776133 10.1111/j.2047-6310.2013.00168.x

[32] Lee MM , Jebb SA , Oke J , Piernas C . Reference values for skeletal muscle mass and fat mass measured by bioelectrical impedance in 390565 UK adults. J Cachexia Sarcopenia Muscle 2020;11:487–496. 31943835 10.1002/jcsm.12523 PMC7113534

[33] Ramírez‐Vélez R , Rincón‐Pabón D , Correa‐Bautista JE , García‐Hermoso A , Izquierdo M . Handgrip strength: normative reference values in males and females aged 6–64 years old in a Colombian population. Clin Nutr ESPEN 2021;44:379–386. 34330493 10.1016/j.clnesp.2021.05.009

[34] Della Peruta C , Lozanoska‐Ochser B , Renzini A , Moresi V , Sanchez Riera C , Bouché M , et al. Sex differences in inflammation and muscle wasting in aging and disease. Int J Mol Sci 2023;24:4651. 36902081 10.3390/ijms24054651 PMC10003083

[35] Lang TF . The bone‐muscle relationship in men and women. J Osteoporos 2011;2011:702735. 22007336 10.4061/2011/702735 PMC3189615

[36] Bohn MK , Horn P , League D , Steele P , Hall A , Adeli K . Pediatric reference intervals for endocrine markers and fertility hormones in healthy children and adolescents on the Siemens Healthineers Atellica immunoassay system. Clin Chem Lab Med 2021;59:1421–1430. 33957708 10.1515/cclm-2021-0050

[37] Manfredo JA , Anand NS , Zemel BS , Kelly A , Magge SN . Body composition and cardiometabolic risk in South Asian adolescents compared with African American and White peers. Diabetes 2021;70.

[38] Huang Y , Gao L , Cheng H , Wang X , Dong H , Yan Y , et al. Difference of glucose and lipid metabolism abnormalities and body fat between the Chinese and USA teenagers. J Glob Health 2023;13:04041. 37199474 10.7189/jogh.13.04041 PMC10193895

[39] Tan ALM , Langley SR , Tan CF , Chai JF , Khoo CM , Leow MK , et al. Ethnicity‐specific skeletal muscle transcriptional signatures and their relevance to insulin resistance in Singapore. J Clin Endocrinol Metab 2019;104:465–486. 30137523 10.1210/jc.2018-00309

[40] Nahon KJ , Kantae V , den Haan R , Hanssen MJW , Harms AC , van der Stelt M , et al. Gene expression of endocannabinoid system components in skeletal muscle and adipose tissue of South Asians and White Caucasians with overweight. Obesity (Silver Spring) 2018;26:1332–1337. 30070030 10.1002/oby.22245

