# Supplementary material for: The life‐course changes in muscle mass using dual‐energy X‐ray absorptiometry: The China BCL study and the US NHANES study
Source: J Cachexia Sarcopenia Muscle. 2024 Jul 1;15(5):1687–95. doi: 10.1002/jcsm.13522 (PMC11446696; doi:10.1002/jcsm.13522)
Supplement: Supplementary file 1 — Table S1. Age composition of the participants Figure S1. Muscle mass index (MMI) (A), appendicular skeletal muscle mass index (ASMMI) (B), arm skeletal muscle mass index (arm‐SMMI) (C) and leg skeletal muscle mass index (leg‐SMMI)(D) reference percentiles for males and females between ages 3 to 65 years in Chinese Figure S2. Comparisons of the 50th percentile curves for muscle mass index (MMI) (A), appendicular skeletal muscle mass index (ASMMI) (B), arm skeletal muscle mass index (arm‐SMMI) (C) and leg skeletal muscle mass index (leg‐SMMI)(D) in males according to age for Chinese versus American (including white, black, and Mexican adults) from NHANES data Figure S3. Comparisons of the 50th percentile curves for muscle mass index (MMI) (A), appendicular skeletal muscle mass index (ASMMI) (B), arm skeletal muscle mass index (arm‐SMMI) (C) and leg skeletal muscle mass index (leg‐SMMI)(D) in females according to age for Chinese versus American (including white, black, and Mexican adults) from NHANES data [file JCSM-15-1687-s001.doc]

**Supplement**

**Table S1** Age composition of the participants

**Fig. S1** Muscle mass index (MMI) (A), appendicular skeletal muscle mass index (ASMMI) (B), arm skeletal muscle mass index (arm-SMMI) (C) and leg skeletal muscle mass index (leg-SMMI)(D) reference percentiles for males and females between ages 3 to 65 years in Chinese

**Fig. S2** Comparisons of the 50^th^ percentile curves for muscle mass index (MMI) (A), appendicular skeletal muscle mass index (ASMMI) (B), arm skeletal muscle mass index (arm-SMMI) (C) and leg skeletal muscle mass index (leg-SMMI)(D) in males according to age for Chinese versus American (including white, black, and Mexican adults) from NHANES data

**Fig. S3** Comparisons of the 50^th^ percentile curves for muscle mass index (MMI) (A), appendicular skeletal muscle mass index (ASMMI) (B), arm skeletal muscle mass index (arm-SMMI) (C) and leg skeletal muscle mass index (leg-SMMI)(D) in females according to age for Chinese versus American (including white, black, and Mexican adults) from NHANES data

**Supplemental Table S1 Age composition of the participants**

|  | **Male** | | | |  | **Female** | | | |
| --- | --- | --- | --- | --- | --- | --- | --- | --- | --- |
|  | Asian Chinese | Non-Hispanic Whites | Non-Hispanic Blacks | Mexican Americans |  | Asian Chinese | Non-Hispanic Whites | Non-Hispanic Blacks | Mexican Americans |
| <18 | 6537 (77.7) | 854 (30.0) | 788 (38.8) | 609 (40.4) |  | 6234 (70.9) | 770 (27.7) | 680 (35.3) | 652 (41.5) |
| 18- | 1009 (12.0) | 600 (21.0) | 440 (21.7) | 332 (22.0) |  | 1363 (15.5) | 577 (20.8) | 381 (19.8) | 294 (18.7) |
| 30- | 627 ( 7.5) | 505 (17.7) | 244 (12.0) | 214 (14.2) |  | 674 ( 7.7) | 481 (17.3) | 227 (11.8) | 202 (12.9) |
| 40- | 167 ( 2.0) | 444 (15.6) | 271 (13.3) | 176 (11.7) |  | 296 ( 3.4) | 482 (17.4) | 322 (16.7) | 238 (15.1) |
| 50-60/59 | 70 ( 0.8) | 448 (15.7) | 287 (14.1) | 175 (11.6) |  | 226 ( 2.6) | 467 (16.8) | 318 (16.5) | 185 (11.8) |

**
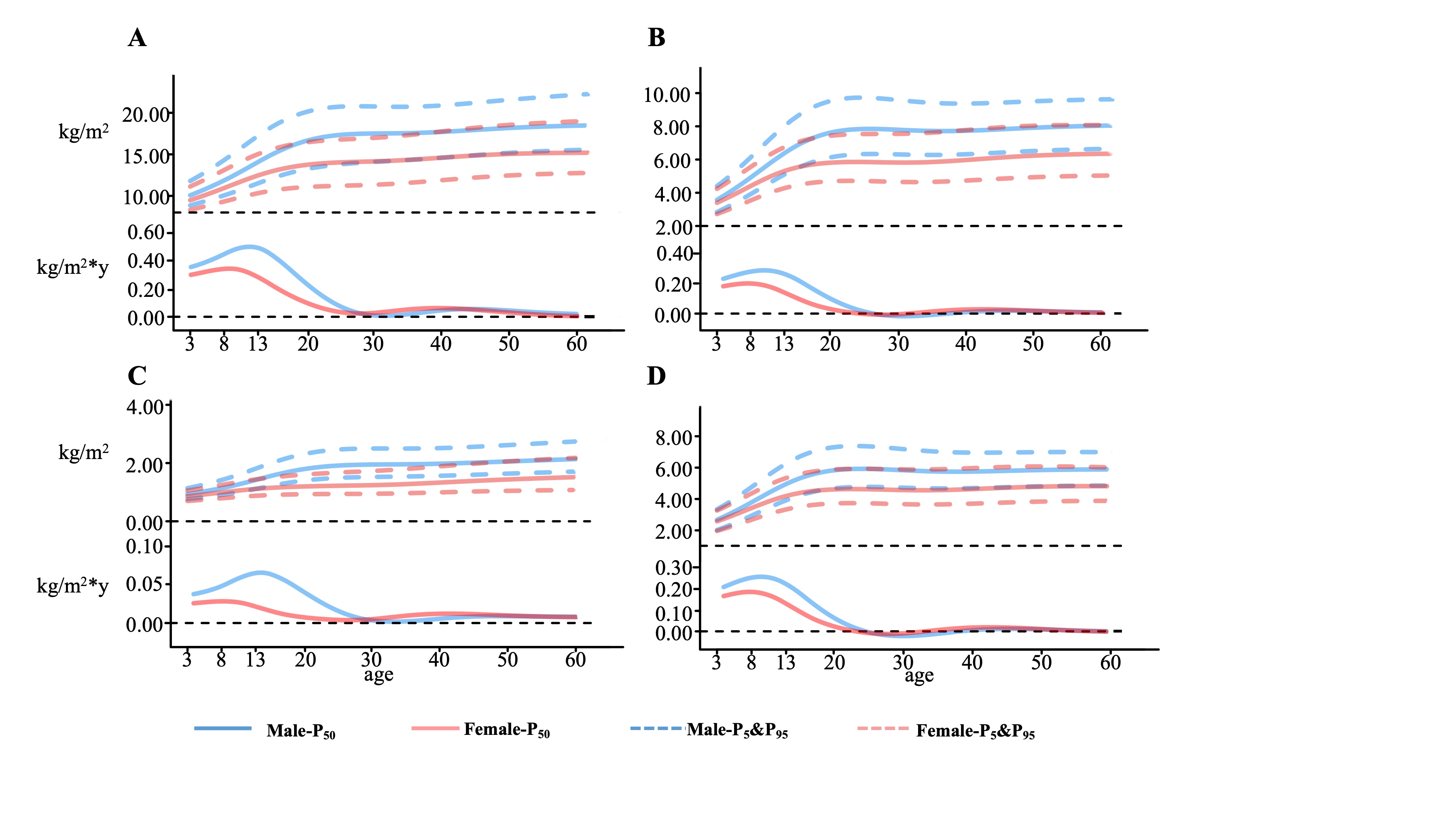
**

**Fig. S1 Muscle mass index (MMI) (A), appendicular skeletal muscle mass index (ASMMI) (B), arm skeletal muscle mass index (arm-SMMI) (C) and leg skeletal muscle mass index (leg-SMMI) (D) reference percentiles for males and females between ages 3 to 60 years in Chinese**

**
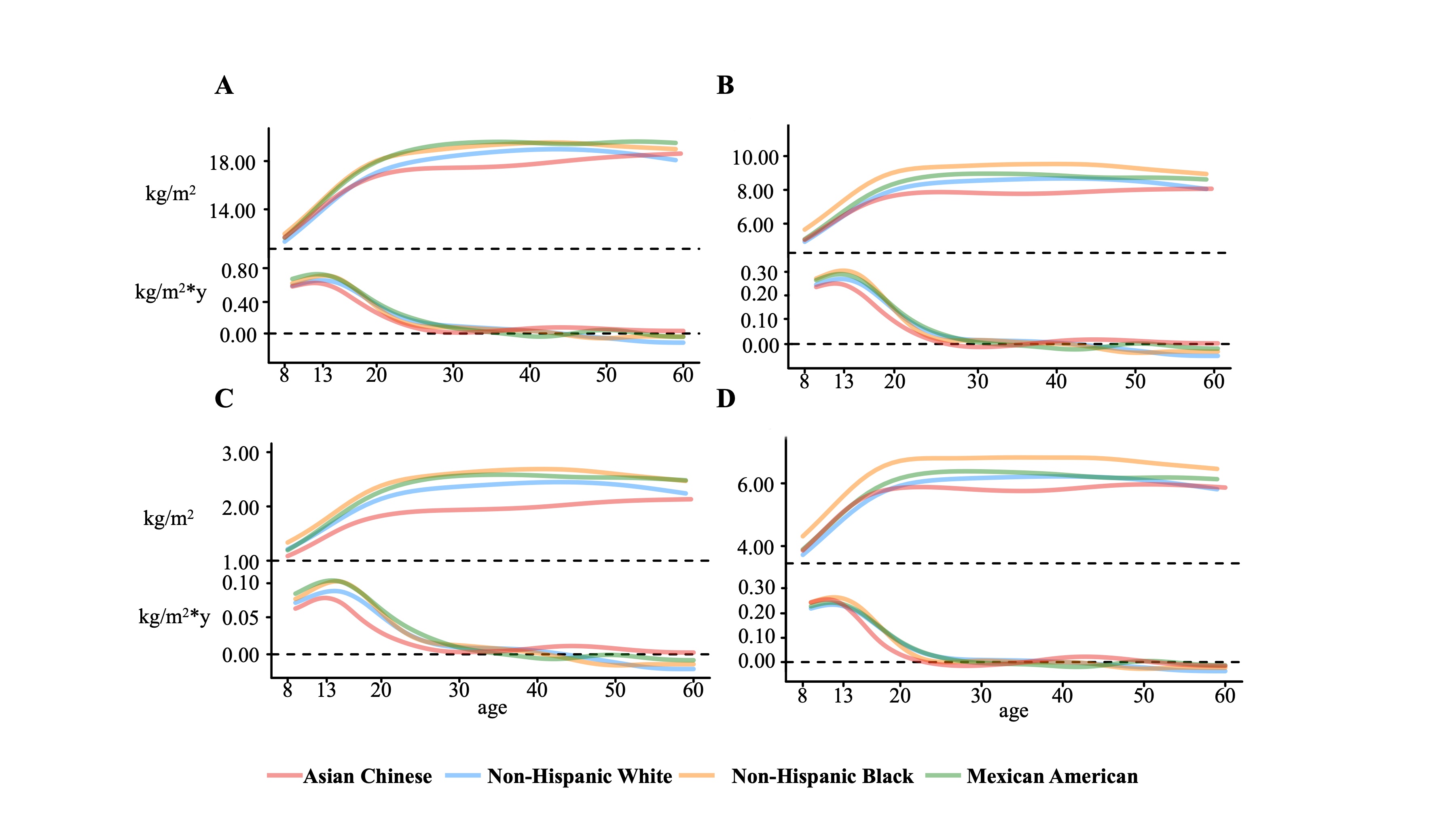
**

**Fig. S2 Comparisons of the 50^th^ percentile curves for muscle mass index (MMI) (A), appendicular skeletal muscle mass index (ASMMI) (B), arm skeletal muscle mass index (arm-SMMI) (C) and leg skeletal muscle mass index (leg-SMMI) (D) in males according to age for Chinese versus American (including white, black, and Mexican adults) from NHANES data**

**
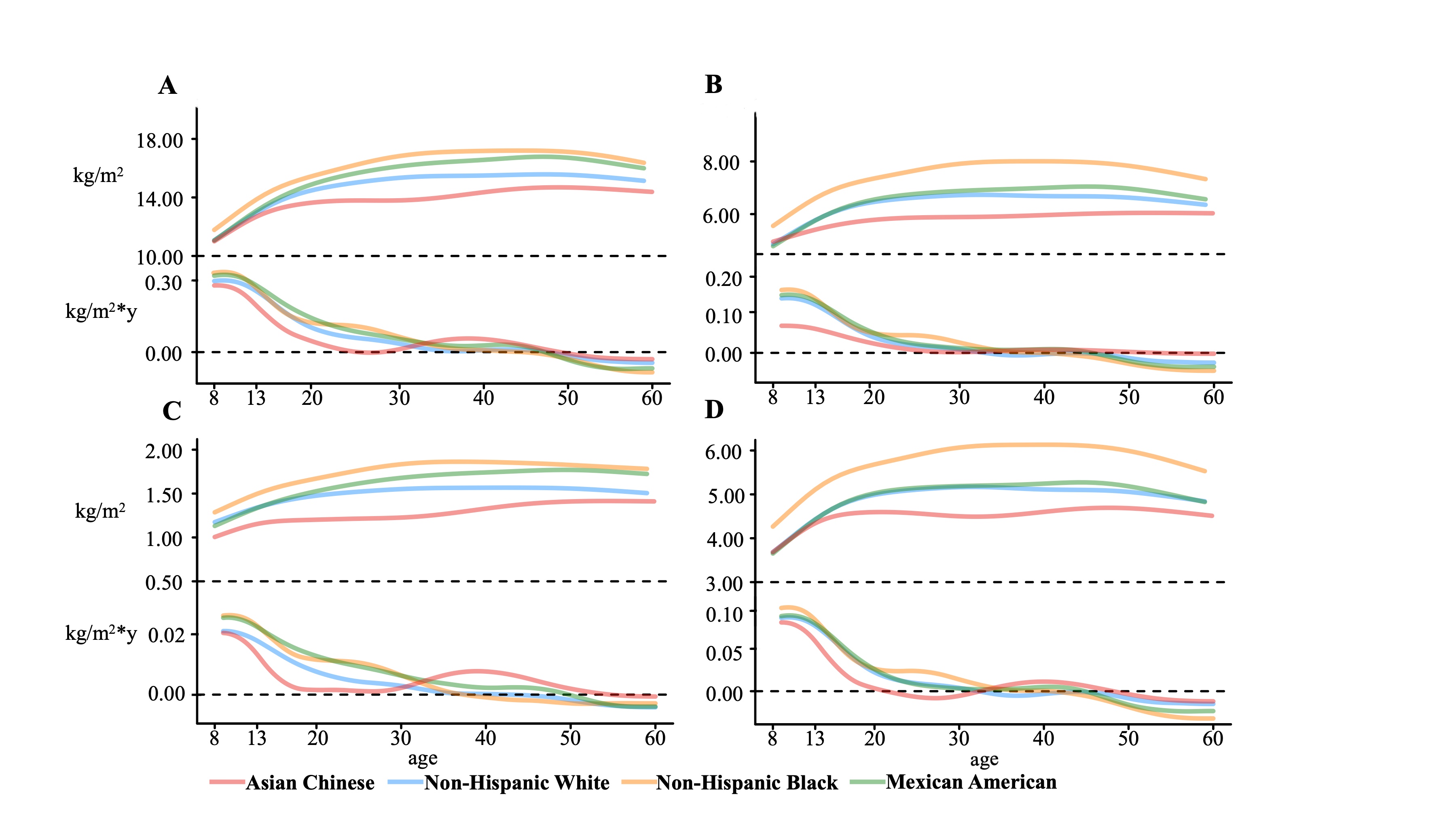
**

**Fig. S3 Comparisons of the 50^th^ percentile curves for muscle mass index (MMI) (A), appendicular skeletal muscle mass index (ASMMI) (B), arm skeletal muscle mass index (arm-SMMI) (C) and leg skeletal muscle mass index (leg-SMMI) (D) in females according to age for Chinese versus American (including white, black, and Mexican adults) from NHANES data**
